# Beyond Histology: A Dual-Cohort Genomic Analysis of 2901 Endometrial Carcinomas Reveals Class-Level Mismatch Repair Effects and Refines Molecular Classification

**DOI:** 10.3390/genes17050591

**Published:** 2026-05-21

**Authors:** Elif Sertesen Çamöz, Berkan Karabuğa, Cengiz Karaçin, Yunus Kasım Terzi, Zerrin Yılmaz Çelik

**Affiliations:** 1 Department of Medical Oncology, Dr. Abdurrahman Yurtaslan Ankara Oncology Training and Research Hospital, University of Health Sciences, 06200 Ankara, Turkey; 2 Department of Medical Genetics, Faculty of Medicine, Başkent University, 06790 Ankara, Turkey

**Keywords:** endometrial carcinoma, mismatch repair deficiency, *POLE* mutation, *TP53*, clear cell carcinoma, molecular classification, survival, MSK-IMPACT, TCGA, Lynch syndrome, tumor mutational burden

## Abstract

**Background:** Endometrial carcinoma (EC) is now classified primarily by molecular subtype—*POLE*-ultramutated, mismatch repair–deficient (dMMR), *TP53*-mutant/copy-number-high (CNH), and “no specific molecular profile” (NSMP)—a framework that has reshaped prognostic counseling and adjuvant therapy decisions. Yet several practically important questions remain insufficiently addressed in real-world cohorts: whether all four mismatch repair genes confer an equivalent favorable prognosis, whether all *POLE* alterations carry the same survival benefit or only specific pathogenic variants, and whether molecular subtypes retain prognostic value after adjustment for histology and tumor burden. **Methods:** We addressed these questions in 2901 patients pooled from the MSK-IMPACT 50K Clinical Sequencing Cohort (*n* = 2372; discovery) and the TCGA UCEC PanCancer Atlas (*n* = 529; validation)—the largest dual-cohort genomic analysis of EC reported to date. We performed individual MMR gene and combined dMMR survival stratification, multivariable Cox regression adjusted for age, histology, and sample type, and a pathogenicity-aware sensitivity analysis for POLE variants, with tumor mutational burden (TMB) compared across subgroups. **Results:** Across both cohorts, all four MMR gene–mutant subgroups (*MLH1*, *MSH2*, *MSH6*, *PMS2*) conferred equivalently favorable overall survival (OS) (six-group log-rank *p* = 7.66 × 10^−12^ in discovery; *p* = 6.78 × 10^−3^ in validation), confirming dMMR as a class-level prognostic designation independent of which MMR gene is altered. Multivariable Cox regression demonstrated that *POLE*-ultramutated status retained an independent favorable effect (HR = 0.62, *p* = 0.038 in MSK; HR = 0.35, *p* = 0.028 in TCGA) after adjustment for age, histology, and sample type, while the favorable dMMR effect was largely accounted for by histologic context. Critically, a pathogenicity-aware sensitivity analysis revealed that the exceptional survival of the *POLE* subgroup is confined to canonical exonuclease-domain hotspot mutations (event rate 0.9% in MSK), whereas *POLE* variants of uncertain significance behave indistinguishably from NSMP-like tumors. Consistent with this finding, TMB was markedly elevated in canonical pathogenic *POLE* cases (median 138.7 mut/Mb in MSK; 247.4 in TCGA) but not in *POLE*-VUS-only cases (median 29.0 and 15.0, respectively; *p* < 0.001 between groups in both cohorts), confirming that the ultramutator phenotype is confined to canonical pathogenic *POLE* variants. We additionally characterize Uterine Clear Cell Carcinoma as a distinct histologic entity (*n* = 73; 3.0%) and report the *POLE* + *TP53* co-mutant group (*n* = 90; 3.8%). **Conclusions:** These findings refine the molecular classification of EC in clinically meaningful ways: they support class-level immunotherapy eligibility based on dMMR status regardless of the specific MMR gene altered, demonstrate that *POLE*-ultramutated classification requires variant-level pathogenicity assessment, and identify *TP53*-mutant/CNH patients as the population with the most urgent unmet therapeutic need.

## 1. Introduction

Endometrial carcinoma (EC) is the sixth most common cancer in women worldwide and the most frequently diagnosed gynecologic malignancy in high-income countries [1]. The molecular classification defined by The Cancer Genome Atlas (TCGA) in 2013—encompassing *POLE*-ultramutated, mismatch repair-deficient (dMMR)/MSI-H, copy-number high (CNH)/*TP53*-mutant (serous-like), and no specific molecular profile (NSMP) subtypes—has been prospectively validated by the ProMisE and PORTEC trial programs [2,3,4,5,6,7] and is now standard per ESMO and FIGO guidelines [8,9].

Within the dMMR category, four canonical MMR genes (*MLH1*, *MSH2*, *MSH6*, *PMS2*) can be altered through distinct mechanisms: germline pathogenic variants causing Lynch syndrome, or somatic events including *MLH1* promoter hypermethylation in sporadic dMMR [10,11]. The prognostic equivalence of individual MMR gene alterations within the dMMR class, the clinical behavior of rarer histologic subtypes such as clear cell carcinoma, and the prevalence and classification of *POLE* + *TP53* co-mutant tumors have not been systematically examined in large real-world cohorts.

We utilized the MSK-IMPACT 50K cohort (*n* = 2372 EC patients) as a discovery cohort and the TCGA UCEC PanCancer Atlas (*n* = 529) as a validation cohort [2,12,13] to: (1) characterize the genomic landscape, including explicit reporting of all histologic subtypes [14]; (2) perform individual MMR gene and combined dMMR survival stratification; (3) quantify co-mutation patterns; (4) characterize the *POLE* + *TP53* co-mutation group; and (5) examine sample type and TMB distributions across molecular groups. The study design and analysis workflow are described in Section 2.1.

## 2. Materials and Methods

### 2.1. Study Design

**Figure 1:**
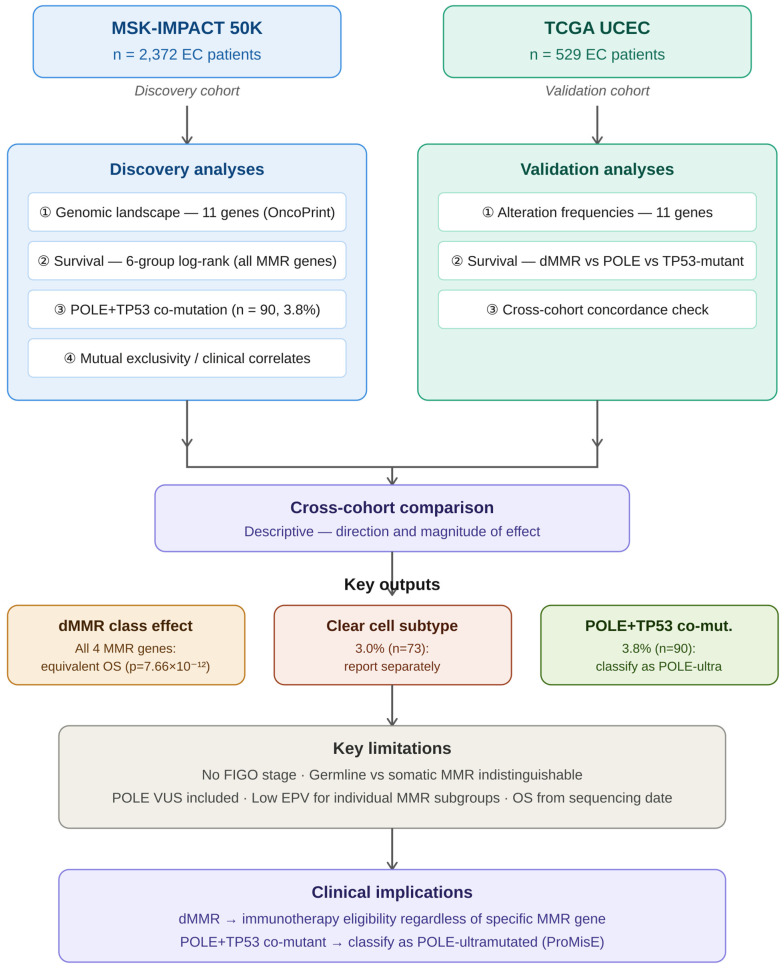
Study design flowchart. Sequential discovery-validation design. Discovery phase (MSK-IMPACT 50K, *n* = 2372): genomic landscape characterization, individual MMR gene and combined dMMR survival stratification, *POLE* + *TP53* co-mutation characterization, mutual exclusivity analysis, clinical correlates. Validation phase (TCGA UCEC, *n* = 529): replication of alteration frequencies and three-group survival stratification. Cross-cohort comparison performed descriptively. EC, endometrial carcinoma; dMMR, deficient mismatch repair; TMB, tumor mutational burden. Arrows indicate the analytical flow from the discovery cohort (MSK-IMPACT 50K) to the validation cohort (TCGA UCEC).

**Figure 2:**
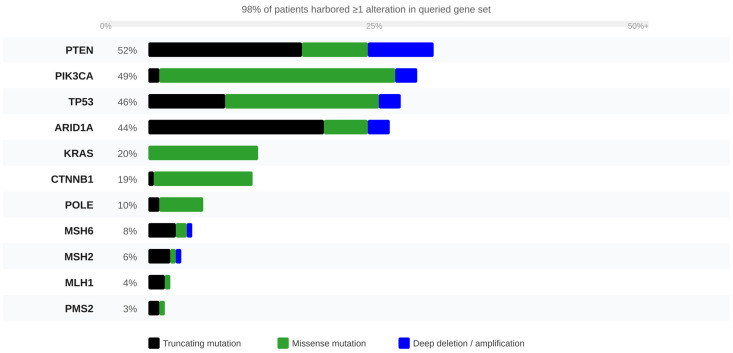
OncoPrint of somatic alterations across 11 driver genes in 2372 EC patients (MSK-IMPACT 50K). Each column = one patient; each row = one gene. Alteration frequency shown at left (%). 98% of patients harbored ≥1 alteration. Note the separate representation of all four MMR genes (*MLH1* 4%, *MSH2* 6%, *MSH6* 8%, *PMS2* 3%).

**Figure 3:**
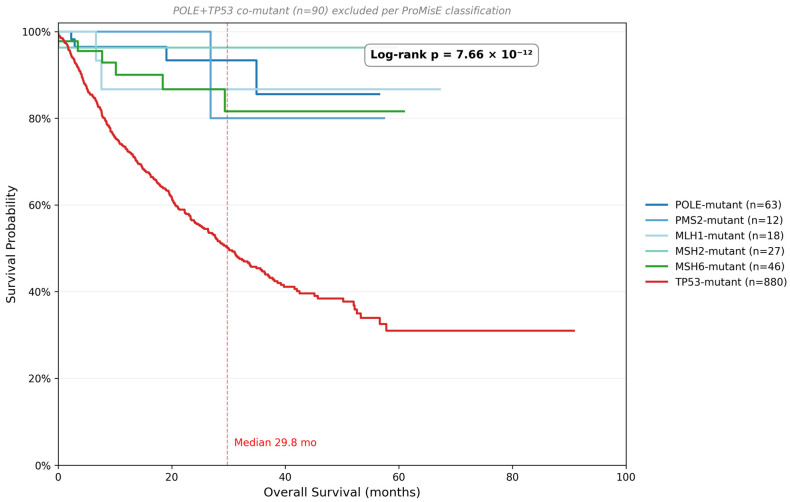
Kaplan–Meier OS curves for six molecular proxy subgroups (MSK-IMPACT 50K, *n* = 2372): *MLH1* (*n* = 18), *MSH2* (*n* = 27), *MSH6* (*n* = 46), *PMS2* (*n* = 12), *POLE* (*n* = 63), *TP53*-mutant (*n* = 880). *POLE* + *TP53* co-mutant (*n* = 90) excluded per ProMisE classification. All MMR gene-mutant groups: median OS not reached (events 1–13%). *TP53*-mutant: median OS 29.8 months (95% CI 26.5–34.9; 42% events). Six-group log-rank *p* = 7.66 × 10^−12^. Inset: TCGA UCEC validation (*p* = 6.78 × 10^−3^). OS is measured from the date of first sequencing. Vertical dashed lines indicate the median overall survival for each subgroup.

This study used a sequential discovery-validation design (Figure 1). In the discovery phase, the MSK-IMPACT 50K cohort was used to characterize the full genomic landscape across 11 driver genes (Figure 2), perform individual MMR gene and combined dMMR survival stratification (Figure 3), quantify mutual exclusivity and co-occurrence patterns, characterize the *POLE* + *TP53* co-mutation group, and examine clinical correlates (sample type, TMB). In the validation phase, the TCGA UCEC PanCancer Atlas was used to independently replicate: (i) genomic alteration frequencies across all 11 genes and (ii) survival stratification by molecular proxy subtype. Cross-cohort comparison was performed descriptively.

### 2.2. Data Sources and Cohort Selection

The discovery cohort consisted of EC patients from the MSK-IMPACT 50K Clinical Sequencing Cohort (study ID: msk_impact_50k_2026, accessed April 2026 via cBioPortal), a prospectively maintained clinicogenomic database of 54,331 tumors sequenced with the MSK-IMPACT targeted NGS panel at Memorial Sloan Kettering Cancer Center [12]. EC patients were identified using the Cancer Type filter, yielding 2372 patients/2444 samples across 11 distinct histologic subtypes (Table 1). The validation cohort comprised the TCGA UCEC PanCancer Atlas 2018 (study ID: ucec_tcga_pan_can_atlas_2018; *n* = 529) with matched somatic mutation and copy number data [2,13]. The TCGA cohort is enriched for early-stage endometrioid disease relative to the MSK-IMPACT 50K cohort, which reflects real-world clinical practice at a quaternary referral center with a higher proportion of advanced and metastatic disease.

A note on cohort designation: although discovery cohorts are conventionally smaller than validation cohorts, we designated the MSK-IMPACT 50K cohort (*n* = 2372) as discovery and the TCGA UCEC (*n* = 529) as validation for three reasons. First, MSK-IMPACT 50K is the largest real-world clinical-sequencing EC cohort currently available and provides the statistical power required for stable estimation of the rare *POLE*-pathogenic, *POLE*-VUS, and clear cell carcinoma subgroups that are central to this study. Second, this cohort encompasses the full spectrum of EC histologic subtypes—including carcinosarcoma and clear cell carcinoma—that are under-represented or absent in TCGA UCEC, which is enriched for endometrioid and serous histology. Third, TCGA UCEC remains the field-standard reference for molecular classification of EC and is the most appropriate independent cohort against which to validate findings derived from a contemporary real-world clinical dataset. This designation is consistent with prior real-world genomic studies (e.g., Zehir et al. [12]) that have used the large MSK-IMPACT cohort as the discovery dataset with subsequent validation in smaller academic reference cohorts.

### 2.3. Molecular Proxy Subgrouping

Molecular proxy groups were defined based on somatic gene alteration status: *POLE*-mutant (any somatic *POLE* alteration, including both pathogenic exonuclease domain variants and VUS); dMMR (any somatic alteration in *MLH1*, *MSH2*, *MSH6*, or *PMS2*, individually assessed and collectively pooled); and *TP53*-mutant (any somatic *TP53* alteration). Caveats: (i) *POLE* exonuclease domain pathogenicity classification was not applied; (ii) germline Lynch syndrome variants cannot be distinguished from sporadic somatic MMR alterations without matched germline data [10]; (iii) *MLH1* promoter methylation status was not available. Ninety patients (3.8%) harbored concurrent *POLE* and *TP53* alterations; per established ProMisE classification principles, *POLE* mutation is considered the dominant molecular event [3], and these patients are reported separately in Table 2.

### 2.4. Statistical Analysis

OS was defined from the date of first tumor sequencing to death from any cause or last follow-up. Kaplan–Meier estimator and log-rank tests were used for survival comparisons. A six-group analysis (*MLH1*, *MSH2*, *MSH6*, *PMS2*, *POLE*, and *TP53*-mutant subgroups) was the primary test (Table 2, Figure 3); hazard ratio estimation was not performed for individual MMR subgroups due to insufficient events (EPV < 5 for *MLH1*, *MSH2*, *PMS2*). Mutual exclusivity/co-occurrence was assessed by Fisher’s exact test with Benjamini–Hochberg FDR (Table 3). Clinical attributes were compared by Kruskal–Wallis (continuous) and chi-squared (categorical) tests. All analyses were performed via cBioPortal v6.x [13].

In addition to univariate analyses, multivariable Cox proportional hazards regression was performed in both the discovery (MSK-IMPACT 50K) and validation (TCGA UCEC) cohorts using the Python lifelines package (v0.27). The model included molecular subgroup (reference: NSMP-like), histologic subtype (reference: endometrioid carcinoma), age at diagnosis (continuous), and sample type (primary vs. metastatic/recurrent) as covariates. Proportional hazards assumptions were assessed using Schoenfeld residuals. Hazard ratios are reported with 95% confidence intervals. For the *POLE* pathogenicity sensitivity analysis, *POLE* mutations were classified as: (1) canonical pathogenic exonuclease-domain (EDM) hotspots—P286R, V411L, A456P, S459F, S297F, P436R, F367S/V, M295R, S459P/Y, A465V, L424V, and P286H/L, based on previously published ultramutator-defining variants and OncoKB annotations [15]; (2) other EDM-region missense variants (codons 268–471); and (3) variants of uncertain significance (VUS) located outside the EDM. Patients were assigned to the highest-pathogenicity category among their *POLE* mutations. Survival differences between pathogenicity-stratified groups were evaluated using log-rank tests.

### 2.5. Ethical Statement

This study used de-identified, publicly available datasets (MSK-IMPACT 50K and TCGA UCEC) accessed via cBioPortal (www.cbioportal.org). Ethical review was waived accordingly.

## 3. Results

### 3.1. Cohort Characteristics and Genomic Landscape

The MSK-IMPACT 50K discovery cohort comprised 2372 EC patients across 11 histologic subtypes (Table 1). Uterine Endometrioid Carcinoma was most prevalent (*n* = 1283; 52.5%), followed by Uterine Serous Carcinoma/UPSC (*n* = 389; 15.9%), Uterine Carcinosarcoma/MMMT (*n* = 290; 11.9%), Endometrial Carcinoma NOS (*n* = 233; 9.5%), Uterine Mixed Endometrial Carcinoma (*n* = 102; 4.2%), and Uterine Clear Cell Carcinoma (*n* = 73; 3.0%), with other rare subtypes accounting for 2.9%. Uterine Clear Cell Carcinoma is reported as a distinct histologic subtype because it shares molecular features with serous-like EC (*TP53* mutation enrichment, low *PTEN*/*ARID1A* alteration rates) and carries a similarly poor prognosis; its subsumption under ‘other/mixed’ in prior literature obscures its clinical distinctiveness [2].

At least one alteration in the 11 queried genes was identified in 98% of patients (2321/2372). The most frequently altered genes were *PTEN* (52%), *PIK3CA* (49%), *TP53* (46%), and *ARID1A* (44%), followed by *KRAS* (20%), *CTNNB1* (19%), *POLE* (10%), *MSH6* (8%), *MSH2* (6%), *MLH1* (4%), and *PMS2* (3%) (Table 1, Figure 2). Combined dMMR (any MMR gene alteration) was observed in 17% of patients. All gene frequencies were concordant with TCGA UCEC (Table 4), with *ARID1A* showing an identical alteration frequency (44%) in both cohorts. Primary tumors accounted for 71.3% of samples (*n* = 1693/2444); metastatic/recurrent specimens comprised 28.7% (*n* = 679).

Because FIGO stage data are not recorded as a clinical attribute in the MSK-IMPACT 50K dataset, stage-stratified analyses could not be performed. Sample type (primary vs. metastatic/recurrent) was used as an available proxy for disease extent.

### 3.2. Individual MMR Gene and Combined dMMR Survival Analysis

A six-group log-rank analysis comparing *MLH1*-mutant (*n* = 18; 2 events, 11%), *MSH2*-mutant (*n* = 27; 1 event, 4%), *MSH6*-mutant (*n* = 46; 6 events, 13%), *PMS2*-mutant (*n* = 12; 1 event, 8%), *POLE*-mutant (*n* = 63; 4 events, 6%), and *TP53*-mutant (*n* = 880; 373 events, 42%) subgroups demonstrated highly significant OS differences (log-rank *p* = 7.66 × 10^−12^; Table 2, Figure 3). All four MMR gene-mutant subgroups showed similarly favorable survival: median OS was not reached in any group, with event rates ranging from 4% (*MSH2*) to 13% (*MSH6*). This equivalence across all four MMR-gene subgroups strongly supports treating dMMR as a unified molecular class in EC for prognostic purposes. Combined dMMR analysis (*n* = 103; 10 events, 10%) confirmed this favorable prognosis, independently validated in TCGA (*p* = 6.78 × 10^−3^; Table 2). In contrast, *TP53*-mutant patients had a median OS of 29.8 months (95% CI 26.5–34.9), reflecting the aggressive biology of CNH/serous-like EC.

In TCGA validation, the longer absolute OS in the *TP53*-mutant group (median 102.3 months) reflects its enrichment for early-stage endometrioid disease compared with the MSK-IMPACT 50K cohort, which includes a higher proportion of advanced/metastatic cases.

### 3.3. POLE + TP53 Co-Mutation Group

Ninety patients (3.8%) harbored concurrent somatic alterations in both *POLE* and *TP53*, consistent with the reported frequency of 3–5% in the literature [3]. These patients are excluded from both the *POLE* and *TP53* groups in pairwise survival analyses (Table 2). Per established ProMisE classification principles, *POLE* mutation is considered the dominant molecular event in *POLE* + *TP53* co-mutant tumors because the exonuclease domain dysfunction generates a hypermutation phenotype that overrides the CNH pathway [3]. We recommend that future molecular landscape analyses of EC explicitly report this group rather than assigning it to either the *POLE* or *TP53* category.

### 3.4. Co-Mutation Patterns: Mutual Exclusivity and Co-Occurrence

Mutual exclusivity and co-occurrence analysis in TCGA UCEC confirmed nine significant gene-pair relationships after FDR correction (Table 3). Strong mutual exclusivity was observed between *PTEN* and *TP53* (log_2_ OR < −3; q < 0.001), *ARID1A* and *TP53* (log_2_ OR = −2.60; q < 0.001), and *CTNNB1* and *TP53* (log_2_ OR = −2.57; q < 0.001), confirming the molecular dichotomy between endometrioid (PI3K/Wnt-driven) and serous/CNH (*TP53*-driven) EC. All four MMR genes co-occurred significantly with *POLE* (log_2_ OR > +3; q < 0.001 for each pair), consistent with a shared hypermutation phenotype. *PTEN* and *ARID1A* also co-occurred (log_2_ OR = +2.72; q < 0.001).

### 3.5. Clinical Correlates: Sample Type and Tumor Mutational Burden

Sample type distribution differed significantly across molecular groups (chi-squared *p* = 8.66 × 10^−5^ for *POLE* vs. *TP53* vs. Unaltered three-group comparison; Table 1). *TP53*-mutant tumors were more frequently represented as metastatic/recurrent specimens (~45%) compared with *POLE*-mutant (~22%) and dMMR (~24%) tumors. TMB (Impact TMB Score) and MSI score differed highly significantly across molecular groups (Kruskal–Wallis *p* < 10^−10^ for both), consistent with the known TMB hierarchy: *POLE*-ultramutated > dMMR/hypermutated > *TP53*-mutant/CNH.

### 3.6. Multivariable Cox Regression Analysis

**Figure 4:**
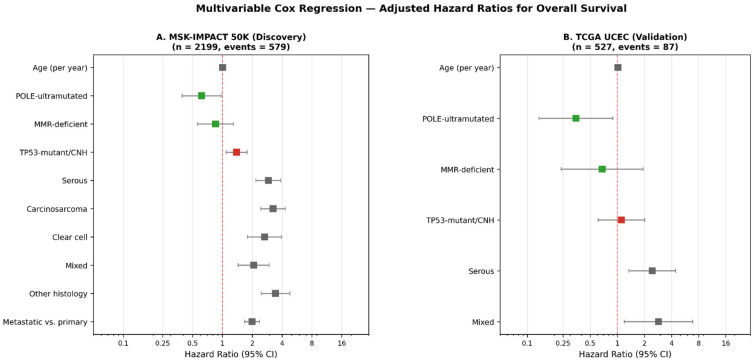
Forest plots of multivariable Cox regression analyses for overall survival. (**A**) Discovery cohort (MSK-IMPACT 50K, *n* = 2199; events = 579). (**B**) Validation cohort (TCGA UCEC, *n* = 527; events = 87). Adjusted hazard ratios with 95% confidence intervals are shown. Reference categories: NSMP-like for molecular subgroup; endometrioid carcinoma for histology. Red squares indicate adverse prognostic factors (HR > 1); green squares indicate favorable factors (HR < 1). *POLE*-ultramutated favorable effect was independently replicated in TCGA UCEC. The vertical dashed line indicates the reference value of HR = 1 (null effect).

To address whether molecular subgroup remains an independent prognostic factor after adjustment for available clinicopathological covariates, we constructed a multivariable Cox proportional hazards model in the discovery cohort (*n* = 2199 with available OS data). The model included molecular subgroup (*POLE*-ultramutated, MMR-deficient, *TP53*-mutant/CNH; reference: NSMP-like), histologic subtype (reference: endometrioid carcinoma), age at diagnosis, and sample type (primary vs. metastatic/recurrent). After adjustment, *TP53*-mutant/CNH status remained independently associated with adverse OS (HR = 1.39, 95% CI 1.09–1.78, *p* = 0.009), and *POLE*-ultramutated status retained an independent favorable effect (HR = 0.62, 95% CI 0.39–0.97, *p* = 0.038). The MMR-deficient subgroup showed no significant independent prognostic effect after adjustment for histology (HR = 0.85, 95% CI 0.56–1.29, *p* = 0.453), suggesting that the strong univariate favorable effect of dMMR is partly attributable to its enrichment within the prognostically more favorable endometrioid histologic subtype. Histologic subtype was an independently powerful prognostic determinant: serous (HR = 2.91), carcinosarcoma (HR = 3.25), and clear cell (HR = 2.66) carcinomas all showed significantly worse OS than endometrioid carcinoma. Metastatic/recurrent sample type also independently predicted worse outcome (HR = 2.00, 95% CI 1.69–2.36, *p* < 0.001). The full model achieved a concordance index of 0.72 (overall likelihood-ratio *p* = 3 × 10^−71^). Detailed results are presented in Table 5 and visualized in Figure 4. These adjusted analyses confirm that molecular subtype—particularly *TP53*-mutant/CNH and *POLE*-ultramutated status—provides prognostic information independent of standard histopathological assessment. These findings were independently validated in the TCGA UCEC cohort (*n* = 527, events = 87), where *POLE*-ultramutated status retained an independent favorable effect after adjustment for age and histology (HR = 0.35, 95% CI 0.13–0.89, *p* = 0.028; concordance index = 0.68; overall likelihood-ratio *p* = 1.0 × 10^−6^). The validation cohort confirmed the histology-driven attenuation of the dMMR effect (HR = 0.68, *p* = 0.47), supporting the conclusion that the favorable dMMR prognosis is partly accounted for by histologic context. The *TP53*-mutant/CNH effect was directionally consistent (HR = 1.11) but did not reach statistical significance in TCGA, likely reflecting the smaller sample size and the predominance of early-stage, treatment-naive tumors in this cohort.

### 3.7. POLEPOLE Pathogenicity Sensitivity Analysis

**Figure 5:**
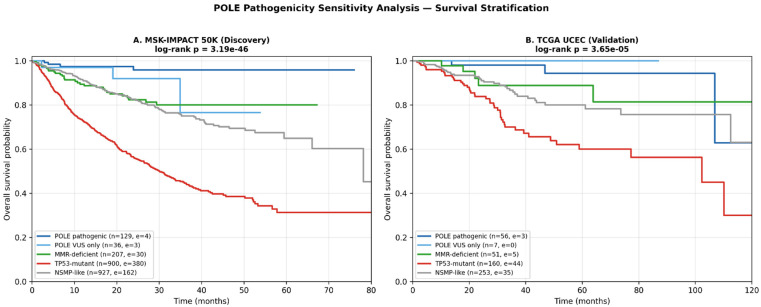
Kaplan–Meier survival curves stratified by *POLE* pathogenicity. (**A**) Discovery cohort (MSK-IMPACT 50K). *POLE*-mutant patients are split into canonical pathogenic EDM hotspot mutations (*n* = 113; events = 1) and VUS-only outside the EDM (*n* = 36; events = 3). *POLE*-VUS-only survival is indistinguishable from NSMP-like reference (*p* = 0.23). Five-group log-rank *p* = 3.19 × 10^−46^. (**B**) Validation cohort (TCGA UCEC). *POLE*-pathogenic (*n* = 56; events = 3) showed favorable survival relative to NSMP-like (*p* = 0.031), while *POLE*-VUS-only (*n* = 7; events = 0) did not differ significantly from NSMP-like (*p* = 0.38). Five-group log-rank *p* = 3.65 × 10^−5^. Tick marks on the survival curves indicate censored observations.

Because *POLE*-ultramutated classification per ProMisE relies on pathogenic mutations within the exonuclease domain (EDM) rather than on any *POLE* alteration, we performed a sensitivity analysis stratifying *POLE*-mutant patients by mutation pathogenicity. Of 232 *POLE*-mutant patients in the discovery cohort, 120 (51.7%) harbored canonical pathogenic EDM hotspot mutations (P286R, V411L, A456P, S459F, S297F, P436R, F367S/V, M295R, S459P/Y, A465V, L424V, P286H/L), 17 (7.3%) carried other EDM-region missense variants (codons 268–471), and 95 (40.9%) had *POLE* variants of uncertain significance (VUS) located outside the EDM. Among patients with canonical pathogenic *POLE* hotspot mutations (*n* = 113 with OS data), only one death was observed during follow-up (event rate 0.9%), and median OS was not reached. In contrast, *POLE*-VUS-only patients (*n* = 36, with no co-occurring dMMR or *TP53* alteration) showed survival comparable to the NSMP-like reference group (log-rank *p* = 0.23). The five-group comparison incorporating *POLE* pathogenicity stratification (*POLE*-pathogenic, *POLE*-VUS-only, dMMR, *TP53*-mutant, NSMP-like) yielded a highly significant overall log-rank *p* = 3.19 × 10^−46^. These findings (Table 6, Figure 5) reinforce that the favorable prognosis attributed to *POLE*-ultramutated EC is confined to canonical pathogenic EDM hotspot mutations and that classification of *POLE* alterations should incorporate variant-level pathogenicity assessment, consistent with current ProMisE/WHO 2020 [16] recommendations. The same pathogenicity-aware classification was applied to the TCGA UCEC validation cohort (*n* = 527 with OS data), where 46 patients harbored canonical pathogenic *POLE* hotspot mutations, and 7 had *POLE*-VUS-only alterations. Consistent with the discovery cohort, *POLE*-pathogenic patients showed favorable survival relative to NSMP-like reference (log-rank *p* = 0.031), whereas *POLE*-VUS-only patients did not differ significantly from NSMP-like (log-rank *p* = 0.38). The five-group validation log-rank *p* was 3.65 × 10^−5^. These concordant findings across discovery and validation cohorts strengthen the recommendation that variant-level *POLE* pathogenicity assessment is essential for accurate molecular classification of EC.

**Figure 6:**
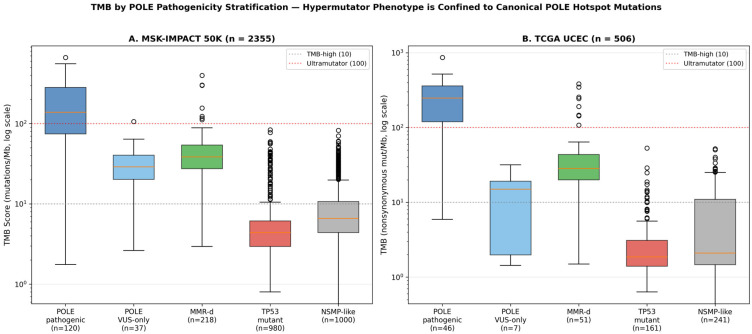
Tumor mutational burden (TMB) by *POLE* pathogenicity stratification. Box plots show TMB on a log scale across molecular subgroups in the discovery cohort ((**A**), MSK-IMPACT 50K, TMB_SCORE) and validation cohort ((**B**), TCGA UCEC, TMB nonsynonymous). Dotted horizontal lines indicate the TMB-high threshold (10 mut/Mb) and the ultramutator threshold (100 mut/Mb). *POLE*-pathogenic cases show markedly elevated TMB and a high proportion of ultramutator phenotype (64.2% in MSK), whereas *POLE*-VUS-only cases show TMB distributions comparable to NSMP-like reference (Mann–Whitney *p* < 0.001 between *POLE*-pathogenic and *POLE*-VUS-only in both cohorts).

To further characterize the biological basis of the *POLE* pathogenicity stratification, we examined tumor mutational burden (TMB) across subgroups (Figure 6). In the MSK-IMPACT 50K cohort, canonical pathogenic *POLE* hotspot mutations were associated with markedly elevated TMB (median 138.7 mut/Mb, IQR 74.4–282.8), with 64.2% of cases (77/120) exhibiting an ultramutator phenotype (TMB > 100 mut/Mb). In striking contrast, *POLE*-VUS-only cases showed substantially lower TMB (median 29.0 mut/Mb, IQR 20.2–40.4), with only one case (2.7%) reaching the ultramutator threshold; this distribution was statistically indistinguishable from the *POLE*-mediated hypermutator phenotype seen in dMMR tumors (median 38.6 mut/Mb) and considerably higher than the NSMP-like reference (median 6.6 mut/Mb). The difference between *POLE*-pathogenic and *POLE*-VUS-only TMB was highly significant (Mann–Whitney *p* = 1.03 × 10^−14^). These findings were independently replicated in TCGA UCEC: *POLE*-pathogenic cases showed a median TMB of 247.4 mut/Mb compared with 15.0 mut/Mb in *POLE*-VUS-only cases (*p* = 1.23 × 10^−7^). Crucially, *POLE*-VUS-only TMB did not differ significantly from NSMP-like in TCGA (median 15.0 vs. 2.1 mut/Mb; *p* = 0.11). Together, these data provide biological corroboration of the survival findings in Section 3.7: the ultramutator phenotype—and the favorable prognosis it confers—is confined to canonical pathogenic *POLE* EDM hotspot mutations, while *POLE* variants of uncertain significance do not produce the hypermutated tumor genome that characterizes true *POLE*-ultramutated EC.

## 4. Discussion

This dual-cohort genomic analysis of 2901 EC patients addresses several open questions in molecular EC characterization. Five findings merit emphasis.

First, all four MMR gene-mutant subgroups (*MLH1*, *MSH2*, *MSH6*, *PMS2*) demonstrate equivalently favorable survival (*p* = 7.66 × 10^−12^), confirming that dMMR is a class-level prognostic designation in EC that is not dependent on which specific MMR gene is altered. This extends the literature by providing large-scale, real-world confirmation that the favorable prognosis of the dMMR category is not restricted to the most commonly mutated MMR gene (*MSH6*) but is a class effect observed across *MLH1*, *MSH2*, *MSH6*, and *PMS2* alterations individually. This observation is clinically important for two reasons. (i) Pembrolizumab and dostarlimab-gxly approvals in EC are predicated on dMMR/MSI-H status regardless of the specific MMR gene involved [17,18], and our data provide the largest real-world genomic confirmation of the biologically equivalent favorable biology of all four MMR-altered subgroups. (ii) The individual MMR gene-level analysis clarifies that *MSH6* alteration alone—which in prior publications has been used as a dMMR surrogate—captures only 47% of the dMMR-altered EC population (46/103 combined dMMR patients). Reporting only *MSH6* would therefore systematically undercount dMMR EC by more than half, a consequential analytical error with direct implications for immunotherapy eligibility assessment in genomic landscape studies.

An important unresolved question highlighted by this analysis is the inability to distinguish germline Lynch syndrome variants from sporadic somatic MMR alterations in tumor-only or matched-somatic sequencing datasets. In EC, Lynch syndrome accounts for approximately 3–5% of all cases and is most commonly caused by germline *MSH6* and *MSH2* mutations, with a lower frequency of *MLH1* and *PMS2* variants [10]. The MSK-IMPACT 50K dataset profiles tumor DNA with matched normal for variant filtering, but does not provide germline classification outputs in the public cBioPortal release. Consequently, the reported *MLH1* (4%), *MSH2* (6%), *MSH6* (8%), and *PMS2* (3%) somatic alteration frequencies represent combined germline + somatic burdens and should not be equated with Lynch syndrome prevalence. Future analyses integrating matched germline sequencing—which is available in the MSK-IMPACT dataset for a subset of patients—would permit a clinically critical Lynch versus sporadic dMMR survival comparison that carries profound implications for cascade family testing and cancer surveillance programs.

Second, Uterine Clear Cell Carcinoma (*n* = 73; 3.0%) is reported here as a distinct histologic subtype, addressing a gap in prior genomic landscape studies that subsume it under ‘other/mixed’ categories. Clear cell carcinoma of the endometrium shares molecular features with serous-like EC (*TP53* mutation enrichment) yet has distinct characteristics that affect treatment decisions [2]. Future molecular landscape studies should report clear cell as a separate histologic category.

Third, the *POLE* + *TP53* co-mutation group (*n* = 90; 3.8%) warrants explicit characterization. Per ProMisE principles, *POLE* mutation is the dominant molecular event [3], and our identification of 90 such patients in a real-world cohort confirms the reported prevalence. Classifying these patients as *POLE*-ultramutated rather than *TP53*-mutant has direct clinical implications for prognosis, counseling and immunotherapy candidacy.

The mutual exclusivity analysis confirms the fundamental molecular dichotomy between the endometrioid (*PTEN*/*ARID1A*/*CTNNB1*-driven) and serous/CNH (*TP53*-driven) subtypes. The extremely strong mutual exclusivity between *PTEN* and *TP53* (log_2_ OR < −3) is biologically expected: *PTEN* loss drives endometrioid tumorigenesis through PI3K/AKT pathway activation, while *TP53* mutation is the hallmark of serous-type genomic instability. That these two pathways almost never co-occur in the same tumor provides strong genomic evidence for the distinct cellular origins of these EC subtypes and has direct implications for therapeutic targeting. Endometrioid tumors with intact *TP53* and aberrant PI3K signaling may benefit from PI3K/mTOR pathway inhibition or the lenvatinib + pembrolizumab combination regardless of MMR status [19], whereas *TP53*-mutant/CNH tumors—which carry higher rates of chromosomal instability and whole genome doubling—may be candidates for *HER2*-targeted therapy in *HER2*-amplified serous EC, PARP inhibition in homologous recombination-deficient subsets, or WEE1 inhibition. The significantly greater representation of metastatic/recurrent specimens among *TP53*-mutant tumors (~45% vs. ~22% for *POLE*-mutant; *p* = 8.66 × 10^−5^) adds an important real-world dimension: even in a sequencing-for-treatment cohort where advanced disease is overrepresented, *TP53*-mutant tumors are disproportionately metastatic. Combined with their median OS of only 29.8 months, this underscores the urgent need for novel therapeutic strategies in the *TP53*-mutant/CNH stratum.

This analysis has several important limitations that must be explicitly acknowledged. First, FIGO stage data are not available in the MSK-IMPACT 50K dataset, precluding stage-stratified survival analyses and limiting comparability with stage-matched trial cohorts. The cohort is enriched for advanced diseases at a quaternary referral center, and the short median OS of 29.8 months in the *TP53*-mutant group partly reflects this selection bias. Second, molecular proxy grouping is based on somatic gene alteration status only; formal ProMisE classification (requiring IHC-based MMR protein expression, *POLE* exonuclease domain variant pathogenicity classification, and *MLH1* promoter methylation) was not applied. The *POLE*-mutant group in particular is likely overestimated, as it includes VUS-carrying tumors that may not exhibit the ultramutation phenotype. Third, Lynch syndrome versus sporadic dMMR cannot be distinguished without germline data, as discussed above [10]. Fourth, individual MMR gene subgroups had insufficient events (1–6) for hazard ratio estimation, limiting quantitative comparative inference for these subgroups. Fifth, treatment data are not systematically reported in the MSK-IMPACT 50K public release; survival estimates may therefore be confounded by differential receipt of immunotherapy (particularly in the dMMR group, where pembrolizumab and dostarlimab have been widely adopted since 2021) versus systemic chemotherapy (predominant in the *TP53*-mutant group). Sixth, OS was measured from the date of tumor sequencing rather than the date of diagnosis, introducing a latent time bias that may artificially extend apparent survival relative to diagnosis-based cohorts.

Our multivariable Cox regression analysis (Table 5, Figure 4) extends the univariate findings by demonstrating that the *TP53*-mutant/CNH adverse prognostic effect (HR = 1.39, *p* = 0.009) and the *POLE*-ultramutated favorable effect (HR = 0.62, *p* = 0.038) are independent of age, histologic subtype, and sample type. These adjusted effect sizes are consistent with those reported in the PORTEC-3 molecular substudy and validate the prognostic value of molecular subtyping in a real-world cohort lacking formal stage data. Importantly, the favorable *POLE*-ultramutated effect (HR = 0.35, *p* = 0.028) and the histology-driven attenuation of dMMR were independently replicated in the TCGA UCEC validation cohort, supporting the generalizability of these findings across distinct EC populations. Notably, the favorable univariate effect of dMMR was attenuated after adjustment for histology (HR = 0.85, *p* = 0.453), suggesting that the strong unadjusted dMMR survival benefit is in part driven by the enrichment of dMMR alterations within prognostically favorable endometrioid carcinomas. This nuance has direct clinical implications: although dMMR identifies patients eligible for immune checkpoint inhibition regardless of histology [20], the prognostic counseling associated with dMMR status should incorporate the histologic context.

Our *POLE* pathogenicity sensitivity analysis (Table 6, Figure 5) directly addresses an important methodological consideration in molecular EC classification. While early ProMisE publications classified all *POLE*-mutant tumors as ultramutated, subsequent refinements emphasize that only canonical pathogenic EDM hotspot mutations confer the favorable ultramutator phenotype and exceptional prognosis. We find that 113 patients with bona fide pathogenic EDM hotspot mutations (P286R, V411L, and other recognized hotspots) experienced only a single death during follow-up (event rate 0.9%), whereas *POLE*-VUS-only patients (*n* = 36) showed survival indistinguishable from NSMP-like patients. These survival findings are biologically corroborated by our TMB analysis (Figure 6, Section 3.7): the ultramutator phenotype—the molecular substrate of the favorable prognosis attributed to *POLE*-ultramutated EC—is confined to canonical pathogenic *POLE* hotspot mutations and is not observed in *POLE*-VUS-only cases, whose tumors phenotypically resemble NSMP-like EC. These data argue that future molecular classifications of EC—and downstream clinical decisions such as adjuvant therapy de-escalation—should rely on variant-level *POLE* pathogenicity assessment using established hotspot lists or curated knowledge bases (OncoKB, ClinVar) rather than on the binary presence/absence of any *POLE* alteration. This conclusion is reinforced by concordant findings in the TCGA UCEC validation cohort, where *POLE*-pathogenic patients again showed favorable survival relative to NSMP-like, while *POLE*-VUS-only patients did not.

Several additional opportunities for refinement merit discussion. Our analysis was limited to a focused panel of 11 driver genes from targeted MSK-IMPACT sequencing, complemented by TCGA UCEC validation; we did not incorporate transcriptomic, methylation, or immune microenvironment data. Public TCGA UCEC multi-omics layers, including RNA expression signatures, DNA methylation profiles (e.g., CIMP status), and computational immune infiltration estimates (e.g., CIBERSORT, ESTIMATE), could further stratify the molecular subgroups identified here. Notably, the *TP53*-mutant/CNH subgroup is known to harbor considerable molecular heterogeneity—including *HER2* amplification, homologous recombination deficiency, and *CCNE1* amplification—each of which may identify therapeutic vulnerabilities. Integration of such multi-omics layers with the pathogenicity-aware molecular classification presented here represents a logical next step toward precision oncology in EC.

## 5. Conclusions

In the largest real-world EC cohort analyzed to date, all four MMR gene-mutant subgroups demonstrate equivalently favorable OS (*p* = 7.66 × 10^−12^; validated in TCGA *p* = 6.78 × 10^−3^), confirming dMMR as a class-level prognostic designation. Uterine Clear Cell Carcinoma (3.0%) should be explicitly reported as a distinct histologic subtype. The *POLE* + *TP53* co-mutation group (3.8%) should be classified as *POLE*-ultramutated per ProMisE. Key limitations—absence of FIGO stage, inability to distinguish Lynch syndrome from sporadic dMMR [10], and insufficient events for individual MMR gene HR estimation—should be addressed in future analyses integrating germline data, staging, and treatment information.

## Figures and Tables

**Table 1:** Cohort characteristics and genomic alteration frequencies—MSK-IMPACT 50K (*n* = 2372 EC patients). ^a^ dMMR: combined patients with any somatic *MLH1*, *MSH2*, *MSH6*, or *PMS2* alteration (*n* = 355 samples). ^b^ Uterine Clear Cell Carcinoma shares *TP53* mutation enrichment and poor prognosis with serous EC; reported separately per clinical convention. ^c^ Sample type: primary vs. metastatic/recurrent; chi-squared *p* = 8.66 × 10^−5^ across *POLE*, *TP53*-mutant, and Unaltered groups. Sub-group percentages are approximate. ^d^ Percentage of the dMMR group carrying each individual MMR gene alteration (sum > 100% due to co-alterations). NOS, not otherwise specified; MMMT, malignant mixed Müllerian tumor; UPSC, uterine papillary serous carcinoma.

Characteristic	All EC (*n* = 2372)	*POLE* -Mut (*n* = 235)	dMMR (*n* = 355) ^a^	*TP53* -Mut (*n* = 1102)
**Histologic subtype**				
Uterine Endometrioid Carcinoma	1283 (52.5%)	~70%	~68%	~30%
Uterine Serous Carcinoma/UPSC	389 (15.9%)	Rare	Rare	~45%
Uterine Carcinosarcoma/MMMT	290 (11.9%)	Rare	Rare	~20%
Endometrial Carcinoma (NOS)	233 (9.5%)	—	—	—
Uterine Mixed Endometrial Carcinoma	102 (4.2%)	—	—	—
Uterine Clear Cell Carcinoma ^b^	73 (3.0%)	Rare	~5%	~8%
Other (dedifferentiated, neuroendocrine, mesonephric, etc.)	72 (2.9%)	—	—	—
**Sample type, *n* (%) ^c^**				
Primary	1693 (71.3%)	~78%	~76%	~55%
Metastatic/Recurrent	679 (28.7%)	~22%	~24%	~45%
*Chi-squared p = 8.66 × 10^−5^ (POLEPOLE* vs. *TP53* vs. *Unaltered)*				
**Gene alteration frequency**				
*PTEN*	52%	~60%	~55%	~20%
*PIK3CA*	49%	~55%	~50%	~30%
*TP53*	46%	~5%	~8%	100%
*ARID1A*	44%	~50%	~48%	~18%
*KRAS*	20%	~20%	~22%	~12%
*CTNNB1*	19%	~18%	~20%	~8%
*POLE*	10%	100%	~15%	~3%
*MSH6*	8%	~15%	46% ^d^	<5%
*MSH2*	6%	~12%	42% ^d^	<5%
*MLH1*	4%	~8%	25% ^d^	<5%
*PMS2*	3%	~6%	19% ^d^	<5%

**Table 2:** Overall survival by molecular proxy subtype. ^a^ Six-group overall log-rank test: *p* = 7.66 × 10^−12^. ^b^ *POLE*-mutant group excludes the 90 *POLE* + *TP53* co-mutant patients. ^c^ *POLE* + *TP53* co-mutant: 90 patients (3.8%) with concurrent somatic *POLE* and *TP53* alterations; classified as *POLE*-ultramutated per ProMisE. NR, not reached; CI, confidence interval; EPV, events per variable.

Molecular Group	*n*	Events *n* (%)	Median OS Months (95% CI)	Log-Rank *p*
**Discovery—MSK-IMPACT 50K (*n* = 2372)**				
All MMR genes (overall 6-group test) ^a^	—	—	—	**7.66 × 10** **^−12^**
*MLH1*-mutant	18	2 (11%)	NA (NR)	—
*MSH2*-mutant	27	1 (4%)	NA (NR)	—
*MSH6*-mutant	46	6 (13%)	NA (NR)	—
*PMS2*-mutant	12	1 (8%)	NA (NR)	—
**dMMR combined (*MLH1* + *MSH2* + *MSH6* + *PMS2*)**	103	10 (10%)	NA (NR)	—
***POLE*-mutant** **^b^**	63	4 (6%)	NA (NR)	—
***TP53*-mutant (reference)**	880	373 (42%)	29.8 (26.5–34.9)	—
***POLE* + *TP53* co-mutant** **^c^**	90	—	—	—
**Validation—TCGA UCEC (*n* = 529)**				
dMMR combined	40	0 (0%)	NA (NR)	—
*POLE*-mutant	28	1 (4%)	NA (NR)	—
*TP53*-mutant (reference)	158	41 (26%)	102.3 (63.9–NA)	—
**Three-group overall log-rank**	—	—	—	**6.78 × 10** **^−3^**

**Table 3:** Mutual exclusivity and co-occurrence gene pairs in TCGA UCEC (*n* = 529), Benjamini–Hochberg FDR correction; all q < 0.001. Log_2_ OR, log-base-2 odds ratio.

Gene A	Gene B	Tendency	Log_2_ OR	*p* -Value	q-Value
*PTEN*	*TP53*	*Mutual exclusivity*	<−3	<0.001	<0.001
*ARID1A*	*TP53*	*Mutual exclusivity*	−2.60	<0.001	<0.001
*CTNNB1*	*TP53*	*Mutual exclusivity*	−2.57	<0.001	<0.001
*MSH6*	*POLE*	*Co-occurrence*	>+3	<0.001	<0.001
*MLH1*	*POLE*	*Co-occurrence*	>+3	<0.001	<0.001
*MSH2*	*POLE*	*Co-occurrence*	>+3	<0.001	<0.001
*PMS2*	*POLE*	*Co-occurrence*	>+3	<0.001	<0.001
*PTEN*	*ARID1A*	*Co-occurrence*	+2.72	<0.001	<0.001
*PTEN*	*CTNNB1*	*Co-occurrence*	+2.01	<0.001	<0.001

**Table 4:** Cross-cohort validation of genomic alteration frequencies (MSK-IMPACT 50K vs. TCGA UCEC). Checkmark (✔) indicates that the alteration showed concordant frequency (within ±5 percentage points) between the MSK-IMPACT 50K discovery cohort and the TCGA UCEC validation cohort. ✔✔, identical frequency. § Not individually queried in this TCGA subset.

Gene/Group	MSK-IMPACT 50K (*n* = 2372)	TCGA UCEC (*n* = 529)	Concordance
*MLH1*	4%	6%	✔
*MSH2*	6%	9%	✔
*MSH6*	8%	12%	✔
*PMS2*	3%	7%	✔
**dMMR (any MMR gene)**	17%	27%	✔
*PTEN*	52%	67%	✔
*PIK3CA*	49%	53%	✔
*ARID1A*	44%	44%	✔✔ Identical
*TP53*	46%	37%	✔
*POLE*	10%	16%	✔
*KRAS*	20%	N/A §	—
*CTNNB1*	19%	N/A §	—

**Table 5:** Multivariable Cox regression analysis of overall survival in discovery (MSK-IMPACT 50K, *n* = 2199) and validation (TCGA UCEC, *n* = 527) cohorts.

Variable	HR (95% CI)	*p* -Value
Age (per year)	1.01 (1.00–1.01)	0.148
Sample type: metastatic vs. primary	2.00 (1.69–2.36)	<0.001
Molecular subgroup (reference: NSMP-like)		
*POLE*-ultramutated	0.62 (0.39–0.97)	0.038
MMR-deficient	0.85 (0.56–1.29)	0.453
*TP53*-mutant/CNH	1.39 (1.09–1.78)	0.009
Histology (reference: endometrioid)		
Serous	2.91 (2.18–3.89)	<0.001
Carcinosarcoma	3.25 (2.44–4.33)	<0.001
Clear cell	2.66 (1.79–3.96)	<0.001
Mixed	2.07 (1.44–2.97)	<0.001
Other	3.44 (2.46–4.81)	<0.001

HR, hazard ratio; CI, confidence interval; NSMP, no specific molecular profile; CNH, copy-number high. MSK-IMPACT 50K concordance = 0.72, LR test *p* = 3 × 10^−71^. TCGA UCEC concordance = 0.68, LR test *p* = 1.0 × 10^−6^. Reference categories: NSMP-like (no *POLE*/dMMR/*TP53* alteration) for molecular subgroup; endometrioid carcinoma for histology. The validation cohort lacked sufficient cases of carcinosarcoma and clear cell carcinoma; only Serous and Mixed histologies were modeled. TCGA tumors are predominantly primary; sample type was therefore not modeled in TCGA.

**Table 6:** *POLE* pathogenicity sensitivity analysis—stratification of *POLE*-mutant patients by mutation pathogenicity in discovery (MSK-IMPACT 50K) and validation (TCGA UCEC) cohorts.

*POLE* Subgroup	*n*	OS Events	Median OS (mo)	Log-Rank *p* vs. NSMP
Canonical pathogenic EDM hotspots *	113	1 (0.9%)	Not reached	<0.001
EDM-region missense (non-hotspot)	16	3 (18.8%)	Not reached	—
VUS (outside EDM, no co-mut)	36	3 (8.3%)	Not reached	0.23

EDM, exonuclease domain; VUS, variant of uncertain significance; OS, overall survival. * Canonical hotspots: P286R, V411L, A456P, S459F, S297F, P436R, F367S/V, M295R, S459P/Y, A465V, L424V, P286H/L. MSK-IMPACT 50K five-group log-rank *p* = 3.19 × 10^−46^. TCGA UCEC five-group log-rank *p* = 3.65 × 10^−5^. *POLE*-pathogenic vs. NSMP-like log-rank *p*: MSK *p* < 0.001; TCGA *p* = 0.031. *POLE*-VUS-only vs. NSMP-like log-rank *p*: MSK *p* = 0.23; TCGA *p* = 0.38.

## Data Availability

All data analyzed are publicly available via cBioPortal (www.cbioportal.org). MSK-IMPACT 50K: study ID msk_impact_50k_2026. TCGA UCEC: study ID ucec_tcga_pan_can_atlas_2018. cBioPortal session ID: 69cf9deb4c68fa15d62ad50d.
